# O_2_, pH, and Redox Potential Microprofiles around *Potamogeton malaianus* Measured Using Microsensors

**DOI:** 10.1371/journal.pone.0101825

**Published:** 2014-07-08

**Authors:** Bin Dong, Ruiming Han, Guoxiang Wang, Xun Cao

**Affiliations:** 1 Jiangsu Key Laboratory of Environmental Change and Ecological Construction, School of Geographical Science, Nanjing Normal University, Nanjing, P. R. China; 2 School of Resource and Environment, Linyi University, Linyi, P. R. China; Mount Allison University, Canada

## Abstract

This study aimed to elucidate the effects of periphyton on the microprofiles of oxygen (O_2_), pH, and oxidation-reduction potential around the stems and leaves of a submerged macrophyte *Potamogeton malaianus* and on the plant growth in the eutrophic shallow Taihu Lake, China. The microprofiles were measured using a motorized microprofiling system equipped with microsensors. The leaf age of the macrophyte and periphyton exerted significant effects on the microprofiles of O_2_, pH, and oxidation-reduction potential. O_2_ concentration and pH increased whereas the oxidation-reduction potential decreased with decreasing distance to the stem/leaf surface. The fluctuation amplitudes of O_2_, pH, and oxidation-reduction potential were the largest in the microprofiles of mature leaves and the lowest in senescent leaves. The periphyton increased the thickness of the broad diffusive boundary layer and fluctuation amplitudes of O_2_, pH, and oxidation-reduction potential. When the periphyton was removed, the thickness of the broad diffusive boundary layer in the microprofiles of stems, senescent leaves, and mature leaves reduced by 29.0%, 49.72%, and 70.34%, and the O_2_, pH, and oxidation-reduction potential fluctuation amplitudes also declined accordingly. Our results suggest that a thick periphyton exerted negative effects on the growth of macrophytes by providing extensive shading and creating a barrier that hindered the transport of dissolved substances such as O_2_, and led to premature decline in macrophytes in the eutrophic Taihu Lake. The consequent implications can help to elucidate the control mechanism of the broad diffusive boundary layer around macrophytes on nutrient cycling in eutrophic waters and to better understand the role of this layer in the Taihu Lake and other similar eutrophic waters.

## Introduction

The boundary layer around submerged macrophytes surfaces plays an important ecological role in macrophyte growth and nutrient transformation in the aquatic environment [1], [2]. Submerged macrophytes provide most of the accessible surface areas, constant survival substrates, and available nutrients for periphyton in eutrophic lakes [3]. Periphyton is an assemblage of algae, bacteria, fungi, animals, inorganic matter, and organic detritus that remains attached to submerged macrophyte surfaces and forms a special bio-water boundary layer [4]. The submerged macrophyte-water boundary layer occupying the key interface of lake ecosystems remarkably influences the productivity and biogeochemical cycles in shallow lake ecosystems [5], [6]. The periphyton biomass, composition, and structure in the boundary layer are known to be affected mainly by factors such as plant species [7], [8], growing stages [9], [10], nutrient load [9], [11], [12], and hydrodynamics [13]. The special boundary layer with a dense periphyton was found to hinder the long-term growth of submerged macrophytes [1], [14], [15], leading to the degradation and even disappearance of submerged macrophytes [16], [17].

Microsensors can be used for measuring dissolved substances with a high temporal (minutes–hours) and spatial resolution, such as those found in the boundary layer, due to their small tip diameter (10–100 µm) [18]. Measurements applying microsensors have been widely performed in sediments [19], [20], biofilms [21], [22], microbial mats [23], roots [24], [25], [26], and leaves of aquatic macrophytes [27], [28], [29] without causing any disturbance to samples or gradients. Several researchers have used oxygen microsensors and found highly dynamic oxygen (O_2_) concentrations in the periphyton community of *Potamogeton crispus* L. and *Littorella uniflora* L. [27], [30]. A pH microsensor showed an increase in the amplitude of pH values with increased periphyton abundance on an artificial plant leaf [31] and revealed the distribution of pH in the external microenvironment of *Fucus vesiculosus* L. under different light conditions [29]. Oxidation-reduction potential (ORP) microsensors revealed the microenvironments at different electrode potentials in a biocathode [21]. Preliminary studies on O_2_ distribution in the boundary layer around submerged macrophytes have indicated a high rate of photosynthetic and heterotrophic process in the microprofiles due to complex assemblages of periphyton; further, there are steep gradients of O_2_ in the microprofiles because of the consumption or production of O_2_ during metabolism in the periphyton [27], [30]. Therefore, the spatial distribution of dissolved substances in an intact boundary layer might be considerably different from that of the overlying water.

Only a few studies have focused on the microprofiles on submerged macrophytes in fresh waters. The O_2_ concentrations were investigated by using microsensors in the boundary layer around submerged macrophyte leaves [27], [30]. Another study measured the pH in the boundary layer around the submerged macrophyte *P. crispus* L.with limited information [27]. Unfortunately, ORP has largely been neglected in the microprofiles around freshwater submerged macrophytes. There is no synchronous study investigating the spatial distribution of O_2_, pH, and ORP in the boundary layer around the stems and leaves of submerged macrophytes in eutrophic shallow freshwater lakes. A comprehensive study on these parameters is thus essential in the boundary layer for deciphering the overall nutrient cycling in shallow lakes and its ecophysiological effects on submerged macrophytes as well as the associated organisms.

*Potamogeton malaianus*, a perennial submerged macrophyte, is the dominant species extensively distributed in the eutrophic Taihu Lake China [32]. According to our field observation, a plenty of periphyton attached on the surfaces of leaves and stems of *P. malaianus*, and there existed variations in the density of periphyton between apical leaves, middle leaves, basal leaves, and stems on the same macrophyte. The older were the leaves, the denser was the periphyton. The suspended particles, bacteria, and microalgae deposited on the surface of *P. malaianus* exerted detrimental impact on the success and growth of the macrophytes [33]–[35]. It has been found that the primary productivity of *P. malaianus* without the periphyton was 16% higher than that of *P. malaianus* with the periphyton in Gonghu Bay, Taihu Lake [34]. Other research also found that the periphyton significantly inhibited the photosynthetic rate in the submerged macrophytes *Ceratophyllum demersum* and *Myriophyllum spicatum* in June, up to 38% and 51% [35], whtich was similar to the trend in *P. malaianus* [33] in Gonghu Bay, Taihu Lake. In neighboring species, periphyton indeed decreased the production of *Potamogeton perfoliatus* by 60–80% in Lake Balaton [15]. While the periphyton and low-light phenomena both reduced the growth and production of the submerged macrophyte *P. perfoliatus* [1]. These findings implicated that the dynamics of dissolved substances in the intact boundary layer of *P. malaianus*. were key for revealing the regulatory mechanism of the periphyton on the growth and productivity. Unfortunately, to date, little is known about the distribution of O_2_, pH, and ORP in the microprofiles around *P. malaianus*, or about the mechanism that drives variables to generate steep gradients and the very chemical dynamic microenvironment at the macrophyte-water interface yet. Many questions arise in this regard: are there significant differences in the distribution of O_2_, pH, and ORP in the microprofiles between different parts (apical leaves, middle leaves, basal leaves, and stems) of the same plant as noted in the periphyton in the eutrophic Taihu Lake? how is the spatial distribution of O_2_, pH, and ORP in the MBL on a cylindrical stem? how does the periphyton affect the distribution of O_2_, pH, and ORP microprofiles around *P. malaianus* collected from Taihu Lake?

In this study, we investigated the O_2_, pH, and ORP microprofiles around the stems and leaves of *P. malaianus* using microsensors, and aimed at revealing (i) the O_2_, pH, and ORP of microprofiles on different parts (apical leaves, middle leaves, basal leaves, and stem) of the same plant; and (ii) the effect of periphyton on the distribution of O_2_, pH, and ORP in the microprofiles. The findings might lead to the elucidation of the mechanism of the effect of periphyton on the growth and decline of submerged macrophytes in eutrophic waters and provide significant insights into the regulatory mechanism of the microprofiles around macrophytes on nutrient cycling in eutrophic waters.

## Materials and Methods

### Ethics statement

No specific permits were required for the described field studies. The study site is not privately owned or protected in any way, and the field studies did not involve endangered or protected species.

### Study sites and sampling

Mature *P. malaianus* were collected in late August 2013 from Gonghu Bay (120.30 ° E, 31.38 ° N) of the eutrophic (total nitrogen (TN), 1.985±0.065 mg·L^–1^; total phosphorus (TP), 0.265±0.015 mg·L^–1^; NH_4_^+^-N, 0.09±0.003 mg·L^–1^) Taihu Lake, China. *P. malaianus* is the dominant species with extensive distribution in Gonghu Bay and West Taihu Lake [36], [37]. There is abundant macrophyte cover (30%) in Gonghu Bay with relatively good water quality (TN, 1.185±0.015 mg·L^–1^; TP, 0.237±0.010 mg·L^–1^; NH_4_^+^-N, 0.08±0.002 mg·L^–1^). Gonghu Bay is a transition region from the algae-dominated state to macrophyte-dominated state in the northeast part of the Taihu Lake. During the time of our study, macrophytes were in the stable growth period. Young leaves (apical leaf, 1–3), mature leaves (middle leaf, 5–7), and senescent leaves (basal yellow leaf) were simultaneously present on *P. malaianus* from August to October. Ten intact, healthy, and mature *P. malaianus* plants were collected for O_2_, pH, ORP, and periphyton measurements. To avoid periphyton sloughing off during transport, we transported the plants and water separately within 3 h after sample collection. The collected *P. malaianus* were placed in an incubator containing ice, and 5,000 mL water was sampled from the top 30 cm of the microprofiles for measurements of O_2_, pH, and ORP around *P. malaianus*.

### Equipment and measurements

The measurements were conducted immediately after the samples arriving at the laboratory. An intact *P. malaianus* was placed in the aquarium containing 3,000 mL *in situ* water. Macrophyte leaves and stems were fastened with staples on agar plates (4%, w/w) and placed in thermostatted water aquarium (20±0.5°C). Measurements were conducted under controlled light of 300 µmol photons·m^–2^·s^–1^ provided using a 150 W fiber-optic halogen lamp (BC-150; Nanjing, China) placed on a laboratory stand.

Clark-type oxygen microsensors (Unisense, Science Park Aarhus, Denmark) with tip diameters of 10 µm were used to measure the O_2_ distribution and dynamics in the microprofiles around the stems and leaves of *P. malaianus*. The methods described by Spilling et al. [29] and Sand-Jensen et al. [27] were used, with modification. The oxygen microsensor was linearly calibrated from signal readings in O_2_-free and air-saturated fresh water at experimental temperature. The microsensor moved vertically via the motorized micromanipulator. A dissecting microscope was used to trace the position of the microsensor tip and define the periphyton surface. In the study, for practical reasons, the position of the outer limit of the diffuse boundary layer was defined as the extrapolation of the linear O_2_ gradient to the bulk concentration. For the macrophytes, the periphyton community represents a further extension of the diffusive boundary layer, which is characterized by non-linear O_2_ gradients due to the production and consumption of O_2_ [27], [38]. The pH microprofileswas measured using a glass pH microsensor with a tip diameter of 10 µm in combination with a simple open-ended Ag-AgCl reference electrode (REF 321; Unisens A/S, Arhus, Denmark). The sensor was linearly calibrated from signal readings in pH standard buffers of 4.00, 6.86, and 9.18 at the experimental temperature. We positioned the pH microsensor as described for the oxygen microsensor.

The ORP was measured using a platinum microelectrode with a tip diameter of 10 µm in combination with a simple open-ended Ag-AgCl reference electrode (REF 321). The microsensor was linearly calibrated from signal readings in quinhydrone redox buffers (pH 4 and 7) at the experimental temperature. The redox microsensor was positioned as described for the O_2_ sensor. Three different points were measured on every leaf or stem with three replicates.

### Calculation of O_2_ flux

O_2_ flux through the diffusive boundary layer between the periphyton-macrophyte association surface and water above was calculated using Fick's first law of diffusion: *J(x)  = * -*φD dC(x)/dx*. Most periphyton examined had low particle density with high internal water content; therefore, we assumed uniform depth distribution of porosity *φ* and a water diffusion coefficient *D*. The porosity *φ* was set at 1.0 (v/v). *D* in water is 2.0 × 10^−5^ cm^2^ s^−1^ at 20°C [27]. The *dx* represents the depth of periphyton-macrophyte association surface. The *dC(x)/dx* is determined from the linear section of O_2_ gradient in the diffusive boundary layer (DBL). *J(x)* is positive in the case of a flux directed toward the bulk water.

Rapid light curves (RLCs) of *P. malaianus* were measured using the pulse amplitude modulated (PAM) fluorometer (Diving-PAM) with 10-s illumination times and intensities increasing in 8 steps [39]. Data were analyzed using software Wincontrol (Walz GmbH, Effeltrich, Germany).

### Periphyton on macrophytes

Periphyton on leaves (young, mature, and senescent) and stems of *P. malaianus* was sampled simultaneously with *P. malaianus*. Each sample consisted of 10 g fresh weight of *P. malaianus.* The leaves or stems were collected from over ten macrophytes. The subsamples with three replicates were transported to the laboratory in glass jars filled with 200-mL distilled water. Periphyton was scraped using a soft brush and rinsed with distilled water. Microscopic observation confirmed that the leaves or stems were visually clean and undamaged. Subsequently, the distilled water containing the scraping and that in which the corresponding subsample was transported were pooled and diluted to 500 mL. The resulting suspension was divided into four equal parts, and two subsamples were vacuum filtered through precombusted and pre-weighed Whatman GF/C filters (for dry mass analysis) and two others through cellulose acetate membrane filters (for chlorophyll a analysis) [9], [12]. Periphyton was collected on precombusted and preweighed Whatman GF/C filters. The dry mass was determined after the filters with periphyton were dried (24 h, 105°C). Ash mass was determined after the filters with periphyton were combusted at 550°C (4 h) in a muffle furnace. The ash-free dry weight (AFDW) was calculated as the difference between dry weight (DW) and ash weight (AW) [9], [12]. The chlorophyll a content was determined using a standard method with acetone as a solvent, according to [40]. The results were calculated per cm^2^ surface of the substratum. The surface area of the cleaned leaves and stems was measured using a conveyor belt-type Licor LI 3000 Areameter.

### Statistical analysis

Data were checked for the assumption of normal distributions and homogeneity of the variances before statistical analyses. Two-way ANOVAwas used for the analysis of DW, AW, AFDW and the chlorophyll a content of the periphyton at the different leaf ages of macrophytes. If the effect was significant, Tukey honestly significant difference (HSD) test was performed with a confidence limit of 95%. All tests were performed using statistical package SPSS 17.0.

## Results

### O_2_ microprofiles

**Figure 1:**
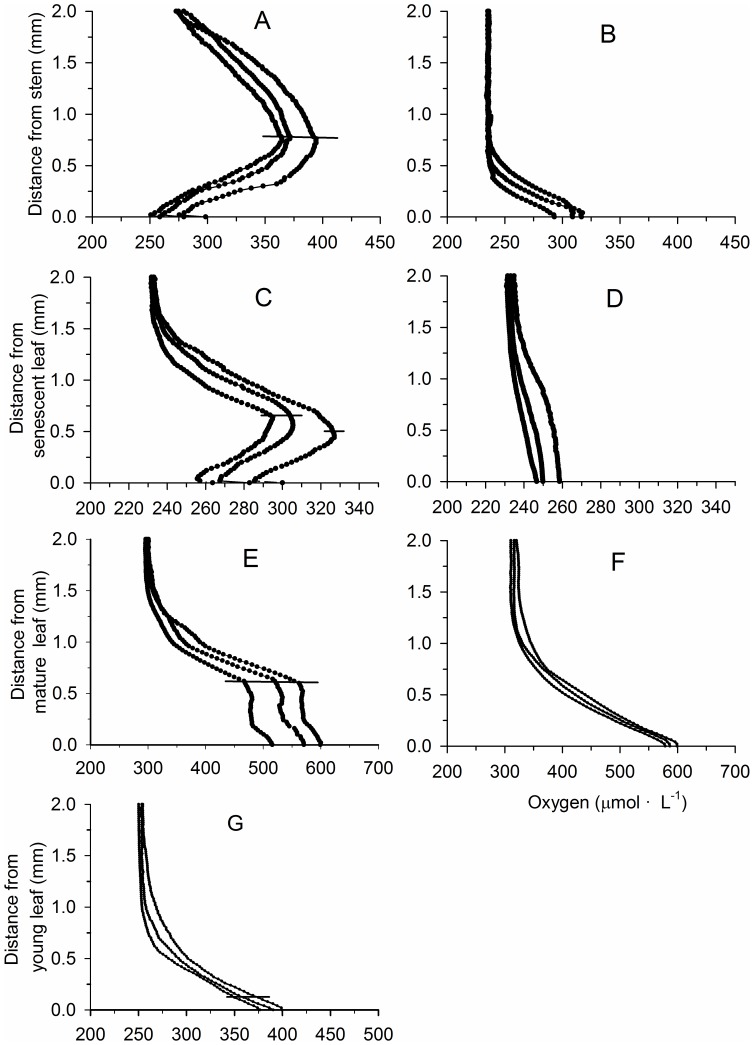
Microprofiles of oxygen around *Potamogeton malaianus* stems and leaves with and without periphyton. A, C, and E indicate thick periphyton; B, D, and F indicate periphyton removed; G indicates young leaves with little periphyton. Microprofiles were measured at three different points under quantum flux density of 300 µmol photons·m^–2^·s^–1^ on *P. malaianus* stems and leaves. The outer surfaces of the periphyton layer are indicated by horizontal bars. The leaf surfaces are indicated by 0. Microprofiles of oxygen around young leaves were not markedly different between the presence of little periphyton and periphyton removed.

O_2_ concentration gradients in the microprofiles significantly changed with decreasing distance from the periphyton surface layer, showing highly diverse patterns among the stems, senescent leaves, mature leaves, and young leaves (Fig. 1A, C, E, and G). O_2_ concentrations increased markedly with decreasing distance from the surface of periphyton in the microprofiles of stems and senescent leaves and peaked at the surface of the periphyton. O_2_ concentrations then gradually decreased from the surface of the periphyton to that of the leaves (Fig. 1A, C). In the microprofiles of mature leaves, O_2_ concentrations was the highest at the surface layer of the periphyton, dropped slightly at the entrance of the periphyton, and then increased gradually and reached the second maximum level at the leaf surface. There was minimum fluctuation in O_2_ concentration gradients in the microprofiles of young leaves (Fig. 1G). O_2_ concentrations in the microprofiles gradually increased with decreasing distance from the leaf surface and reached the maximum at the leaf surface (Fig. 1G). When the periphyton was removed, O_2_ concentrations fluctuation decreased significantly in the microprofiles of the leaves and stems (Fig. 1B, D, F), and the diffusion boundary layer reduced markedly; therefore, the O_2_ concentration decreased significantly at the leaf/stem surface.

**Figure 2:**
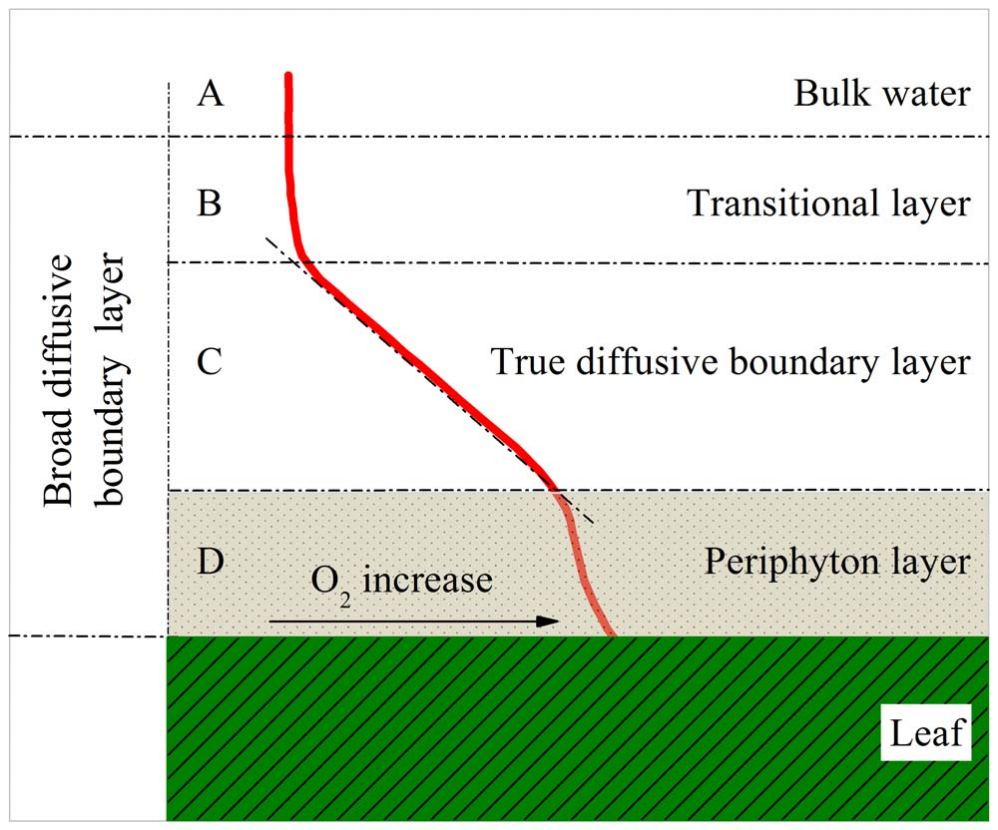
Schematic of oxygen microprofiles around submerged macrophyte leaves with periphyton.

The oxygen microsensor revealed that oxygen microgradients (Fig. 2), the oxygen microgradients showed a transitional layer between the bulk water and the region of molecular oxygen diffusion (Fig. 2B), which showed non-linear O_2_ gradients. The outer limit of the transitional layer was set by the initial deviation of O_2_ concentration relative to the constant bulk water concentration [38].The true DBL (Fig. 2C) was defined by a linearly diffusive gradient, in which oxygen was neither consumed nor produced. However, such linear gradients are usually poorly defined in literature. A more precise and practical definition is obtained from the extrapolation of the linear gradient to the transitional layer water [38]. For the macrophyte, the periphyton layer represented a further extension of the DBL, characterized by non-linear O_2_ gradients due to the production and/or the consumption of O_2_ (Fig. 2D). The thickness of the periphyton layer is usually depends on plant species, growth stages, nutrients load, and hydrodynamics. The integration of layers B, C, and D together were defined as the broad DBL in order to easily describe the micro-spatial distribution of O_2_. Therefore, the broad DBL of O_2_ around submerged macrophytes was not characterized by simple linear O_2_ gradients due to the biological activity of periphyton and macrophytes.

The broad DBL in the microprofiles with periphyton was markedly different from that of the DBL in the microprofiles without the periphyton. When the periphyton was present on the *P. malaianus* leaves or stems, the thickness of the broad DBL in the microprofiles of stems, senescent leaves, and mature leaves was 1701±51 µm, 1160±53 µm, and 1753±27 µm, respectively (extrapolated from the four light profiles of O_2_ measured at different points; Fig. 1A, D, G). O_2_ concentrations at the surface of stems, senescent leaves, and mature leaves were 262.2±19.9 µmol·L^–1^, 282.1±18.2 µmol·L^–1^, and 561.6±42.4 µmol·L^–1^ (Fig. 1), respectively. When the periphyton was removed, the thickness of the DBL in the microprofiles of stems, senescent leaves, and mature leaves was reduced to 493±30 µm, 577±68 µm, and 1233±58 µm (Fig 1C, F, I; Table 1), respectively. The O_2_ concentrations at the surface of stems, senescent leaves, and mature leaves were 301.8±13.5 µmol·L^–1^, 243.7±7.1 µmol·L^–1^, and 518.8±11.6 µmol·L^–1^, respectively (Fig. 1C, F, I; Table 1).

**Table 1:** The characteristics of periphyton on the leaves and stems of *Potamogeton malaianus*.

		Young leaf	Mature leaf	Senescent leaf	Stem
Periphyton	DW (mg·cm^–2^)	0.68±0.04	3.25±0.17	4.72±0.25	5.60±0.30
	AW (mg·cm^–2^)	0.54±0.04	2.51±0.14	3.68±0.19	4.16±0.23
	AW (%)	79.37	77.15	78.02	74.42
	FADW (mg·cm^–2^)	0.14±0.01	0.74±0.04	1.04±±0.06	1.44±0.08
	FADW %	20.63	22.85	21.98	25.58
	Chl a (μg·cm^–2^)	2.54±0.12	12.20±0.73	18.30±0.93	22.20 ± 1.23
	Thickness (μm)	80±7	606±12	623±31	773±41
	Broad DBL (μm)	950±17	1753±33	1160±53	1701±56
Leaf	Length (cm)	12.44±3.70	28.50±4.10	23.00±3.10	-
	Width (cm)	1.01±0.20	1.30±0.20	1.30±0.10	-
	Chl (mg·g^−1^)	0.94±0.05	1.07±0.05	0.51±0.03	-

Values represent means of triplicates and standard error, respectively. DW, dry weight;AW, ash weight; FADW, free ash dry weight; Chl a, chlorophyll a; DBL, diffusive boundary layer

The estimated O_2_ fluxes (J) through the broad DBL varied with leaf age (Table 2). The O_2_ fluxes were the highest in DBL of mature leaves with periphyton removed, and the lowest in the DBL of senescent leaves. The DBL of mature leaves and stems with periphyton had lower O_2_ fluxes than that of mature leaves and stems with periphyton removed. This was because the dense periphyton attached on the surface of mature leaves and stems increased the transport resistance of O_2_.

**Table 2:** The estimated O_2_ fluxes (μmol·cm^–2^·min^–1^) through the broad diffusive boundary layer associated to the surface of *Potamogeton malaianus* according to Fick's first law (n = 3)

	Young leaf (μmol·cm^–2^·min^–1^)	Mature leaf (μmol·cm^–2^·min^–1^)	Senescent leaf (μmol·cm^–2^·min^–1^)	Stem (μmol·cm^–2^·min^–1^)
With periphyton	2.04±0.10	3.02±0.15	1.08±0.05	1.10±0.05
Periphyton removed	NA	3.54±0.18	0.19±0.01	1.36±0.06

Values represent means of triplicates and standard error, respectively. NA, non-applicable.

### pH microprofiles

**Figure 3:**
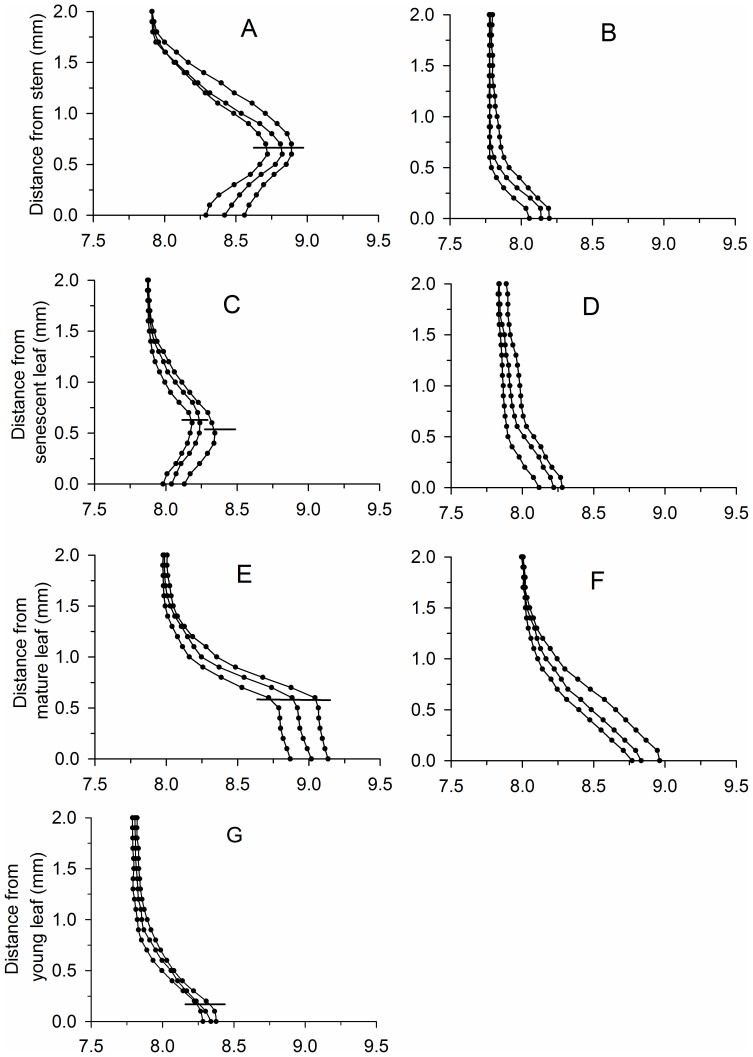
Microprofiles of pH around *Potamogeton malaianus* stems and leaves with and without periphyton. A, C, and E, indicate thick periphyton; B, D, and F indicate periphyton removed; G indicates young leaves with a little periphyton. Microprofiles were measured at three different points under quantum flux density of 300 µmol photons·m^–2^·s^–1^ on *P. malaianus* stems and leaves. The outer surfaces of the periphyton layer are indicated by horizontal bars. The leaf surfaces are indicated by 0. The microprofiles of pH around young leaves were not markedly different between the presence of little periphyton and periphyton removed.

The pH was higher in the microprofiles of *P. malaianus* than in the surrounding water, and the difference varied with leaf age and periphyton layer thickness (Fig. 3). In general, the pH gradients in both length and amplitude increased with decreasing distance from the periphyton layer surface. The trends of change in pH gradients in the microprofiles were the same as those of O_2_ (Fig. 3A, C). The pH increased markedly with decreasing distance from the surface of the periphyton in the microprofiles of stems and senescent leaves and reached the peak at the surface of the periphyton. Subsequently, the pH gradually decreased from the surface of the periphyton to that of the leaves (Fig. 3A, C). In the microprofiles of mature leaves, pH increased to the first peak at the surface layer of the periphyton, dropped slightly after entering the periphyton, and then increased gradually and reached the second peak at the leaf surface. There was minimum fluctuation in pH gradients in the microprofiles of young leaves (Fig. 3G). The pH gradients in the microprofiles gradually increased with decreasing distance from the leaf surface and reached the maximum at the leaf surface (Fig. 3G). When the periphyton was present on *P. malaianus* leaves or stems, the pH at the surface of stems, senescent leaves, and mature leaves was 8.48±0.13, 8.12±0.09, and 9.01±0.13 (Fig. 3), respectively. When the periphyton was removed, pH at the surface of stems, senescent leaves, and mature leaves was 8.13±0.07, 8.20±0.08, and 8.86±0.10, respectively (Fig. 1C, F, I; Table 1), and the pH fluctuation decreased significantly in the microprofiles of leaves and stems (Fig. 3B, D, F), as well as the DBL reduced markedly.

### ORP microprofiles

**Figure 4:**
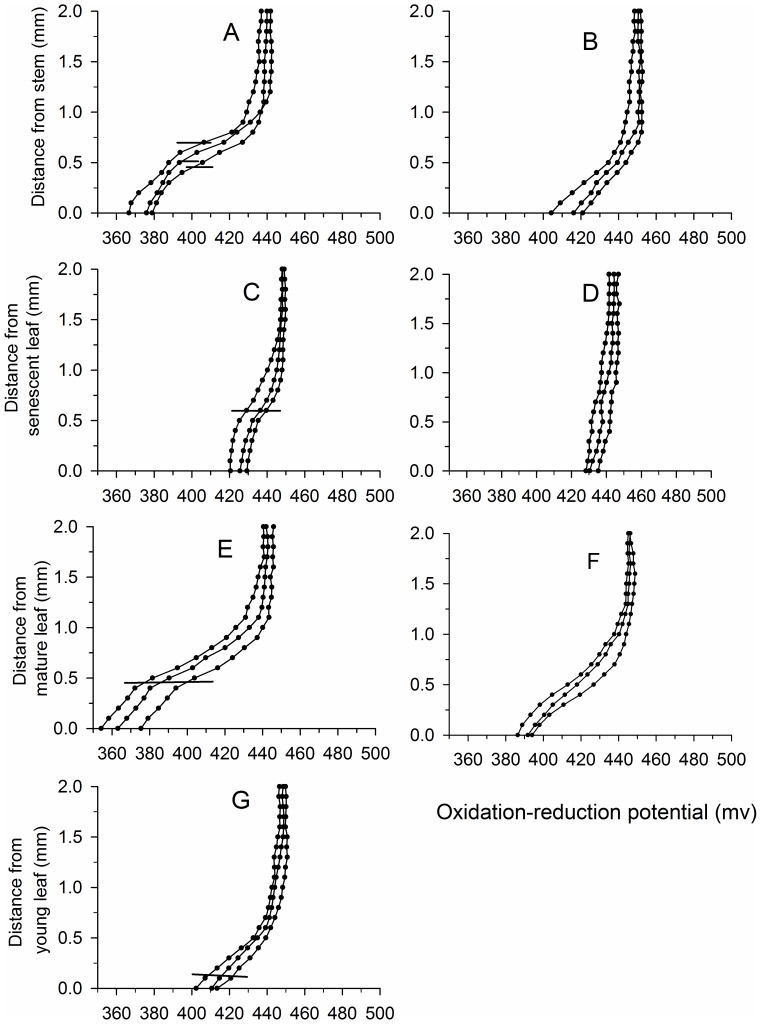
Microprofiles of oxidation-reduction potential around *Potamogeton malaianus* stems and leaves with and without periphyton. A, C, and E, indicate thick periphyton; B, D, and F indicate periphyton removed; G indicates young leaves with little periphyton. Microprofiles were measured at three different points under quantum flux density of 300 µmol photons·m^–2^·s^–1^ on *P. malaianus* stems and leaves. The outer surfaces of the periphyton layer are indicated by horizontal bars. The leaf surfaces are indicated by 0. Microprofiles of oxidation-reduction potential around young leaves were not markedly different between the presence of little periphyton and periphyton removed.

On contrary to micriprofiles of the O_2_ and pH, the ORP markedly decreased with reducing distance from the leaf/stem surface and reached the lowest at the leaf/stem surface. According to leaf age (Fig. 4C, E, G), the fluctuation of ORP in both length and amplitude was the greatest in the microprofiles of mature leaves, followed by that in stems, and was the lowest in the senescent leaves. The periphyton had a visible effect on the distribution of ORP in the microprofiles that, when the periphyton was removed, the ORP fluctuations declined (Fig. 4).

## Discussion

### Leaf age diversifies O_2_, pH, and ORP microprofiles

**Figure 5:**
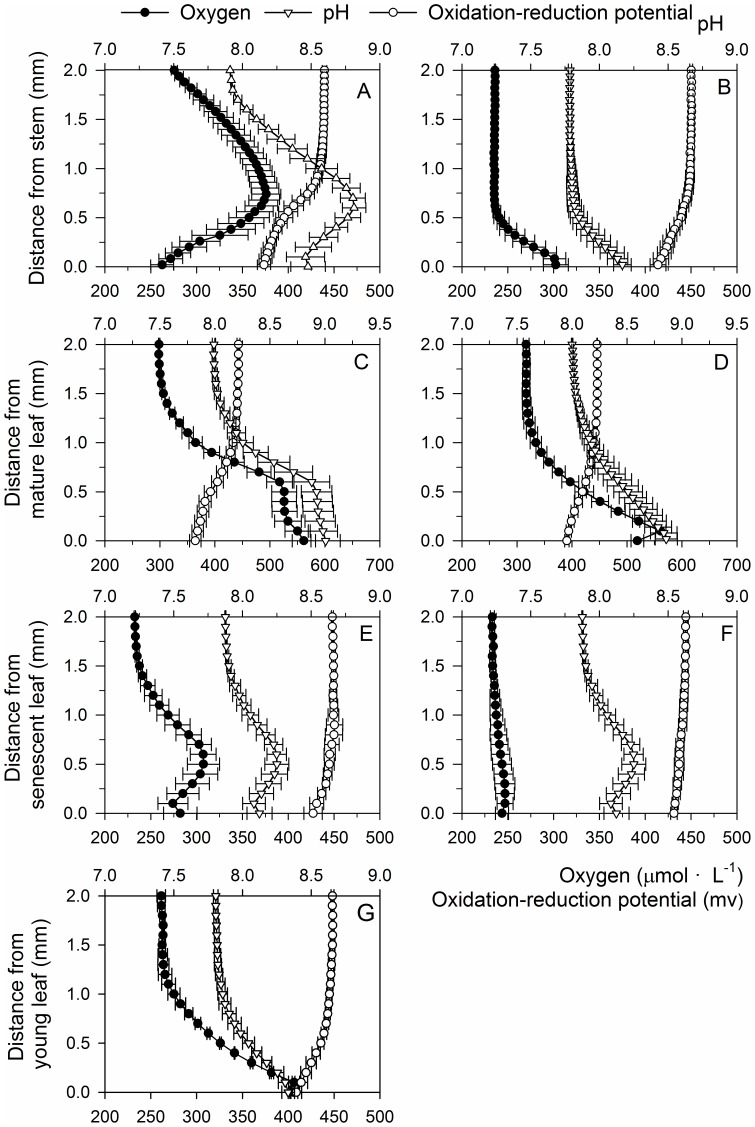
Microprofiles of oxygen, pH, and oxidation-reduction potential around the same *Potamogeton malaianus* stem and leaf. A, C, and E indicate thick periphyton; B, D, and F indicate periphyton removed. Microprofiles were measured under quantum flux density of 300 µmol photons·m^–2^·s^–1^ on *P. malaianus* stems and leaves. Values with bars indicate standard deviations (n = 3).

**Figure 6:**
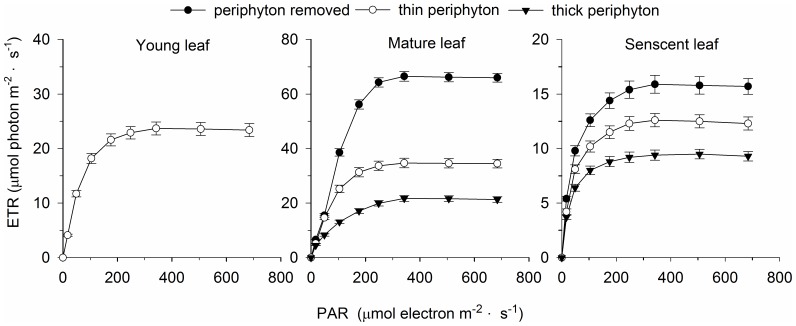
Rapid light curves of *Potamogeton malaianus* with different biomass densities of periphyton. Values with bars indicate standard deviations, n = 3.

The leaf age of the same macrophyte exerted significant effects on the distribution of O_2_ and pH in the microprofiles of *P. malaianus* (Figs. 1, 3, 4, 5). Young leaves used in the present study referred to the first three leaves at the apex of *P. malaianus* since successively younger leaves are always located near the apical meritems. Because the chlorophyll a content, photosynthetic activity, oxygen-release capacity, and periphyton biomass of young leaves were lower than those of mature leaves (Table 1), a thiner DBL was formed, and thus the oxidation microenvironment was weak with less fluctuation of O_2_, pH, and ORP (Figs. 1G, 3G, 4G, 5). Mature leaves possess high chlorophyll a content and photosynthetic activity (Fig. 6), and the barrier formed by dense periphyton led to a strong oxidation microenvironment in the microprofiles. Therefore, O_2_ and pH reached the maximum and ORP reached the minimum at the surface of mature leaves (Figs. 1E, 3E, 4). In this study, senescent leaves were considered the yellowish leaves present at the middle-lower part of *P. malaianus* with the lowest chlorophyll a content and photosynthetic capacity (Table 1, Fig. 6). The periphyton remained attached on the surface of senescent leaves for a long time and formed a thick layer, such that algae could be confirmed by visible inspection on the surface of the thick periphyton layer. Hence, the O_2_ and pH reached the maximum near the surface of the thick periphyton. The consumption of organic matter and utilization of oxygen by heterotrophic organisms may create micro-environments in which the O_2_ and pH decreased markedly (Figs. 1C, 3C, 5). Stems (diameter, 2–5 mm) were 15–30 cm from the shoot tips and had mature leaves and senescent leaves. Due to the relatively low photosynthetic activity of the stem, the trends of changes in O_2_, pH, and ORP were similar to those of senescent leaves. In this study, the O_2_ distribution in the microprofiles of mature leaves was similar to that of *P. crispus* exposed to the light of 318 µE·m^–2^·s^–1^ and *L. uniflora* (L.) exposed to the light of 330 µE·m^–2^·s^–1^ in early June [27].

### The periphyton directly affects O_2_, pH, and ORP

Periphyton adversely affected the rapid light curves of the submerged macrophyte *P. malaianus* (Fig. 6). The light saturation intensity of mature and senescent leaves with the periphyton removed (342.0 µmol·m^–2^·s^–1^) was significantly lower than that of mature and senescent leaves with a thick periphyton layer (506.0 µmol·m^–2^·s^–1^). This suggested that the photosynthetic capacity of leaves with a dense periphyton was lower than that of leaves with periphyton removed.

The periphyton layer combined with the DBL above the surface of the periphyton togetherly formed a thick layer. For the submerged macrophyte *P. malaianus*, this thick layer could be considered as a single thick DBL, through which dissolved substances are transported via molecular diffusion [27]. The distance and resistance of transporting dissolved substances increased at the macrophyte surface owing to the existence of the periphyton layer, which stimulated the thickness of the broad DBL (Table 1) [41], [42]. High O_2_ concentration and high pH value and low ORP were therefore greatly maintained at the leaf surface that had a dense periphyton [6], [27], [43]. When the periphyton was removed, the broad DBL was reduced. In this case, the resulting lower diffusion resistance could not be sustained since high O_2_ concentrations and pH value were maintained at the macrophyte surface as would have been in the presence of the periphyton (Figs. 1, 3, 5) [27]. In our study, O_2_ and pH in the microprofiles at stems and leaf surfaces either with or without the periphyton layer were lower than that reported for *P. crispus* perviously [27]. This could partly attributed to the lower thickness of the periphyton (80.3±6.6 µm to 623.3±30.6 µm) and the higher experimental temperature (20 °C) which were both demonstrated in our study to lower the oxygenation and alkalinity [27]. Regarding the high diversity among macrophyte species, the thickness of the broad DBL of stems, senescent leaves, and mature leaves in *P. malaianus* was considerably higher than those in the brown alga *F. vesiculosus* L. (broad DBL less than 500 µm) [29], in which the presence of little periphyton on the surface of might induce significant difference in the layer's thickness.

The distribution of O_2_, pH, and ORP in the microprofiles around *P. malaianus* also depended on microorganism density and the proportion of photoautotrophic vs. heterotrophic organisms in the periphyton [44]. In this study, the chlorophyll a content was higher (18.3±0.9 µg·cm^–2^ and 22.2±1.2 µg·cm^–2^) in the periphyton separated from senescent leaves and stems than that of from mature and young leaves (Table 1), which led to the highest O_2_ concentration and pH near the surface of the periphyton. However, the marked decrease in O_2_ and pH within the periphyton of senescent leaves could be explained by the low photosynthetic activity (Fig. 6). The pH had been shown to increase in both length and amplitude with increasing periphyton abundance on artificial plant substrates [31]. The reported fluctuation [31] was the same as that for mature leaves and evidently different from that of stems and senescent leaves (Fig. 3). This discrepancy could be attributed to the fact that the periphyton was mainly composed of algae and neutral artificial substrates in the previous study, however, a large fraction of the periphyton was inorganic matter (ash weight) in our study (Table 1).

ORP decreased markedly with reducing distance from the leaf/stem surface (Fig. 4). ORP was the lowest near the surface of mature leaves and highest near the surface of senescent leaves (Figs. 4, 5). The ORP is used to access the tendency to donate or receive electrons and is a combined result of redox by various oxidizing and reducing substances. Although it is not a direct measure of the concentrations of these substances, it can help understand the electrochemical characteristics of a water body and can be used as a quantitative measure to characterize the relative extent of oxidation in a lake [45]. The concentration of oxides and reduction in pH and temperature are the main factors that affect the ORP in water. Nernst equation is a linear equation for ORP (*E_h_*) as a function of pH with a slope of -0.05916*h/n* volt. This equation predicts lower ORP at higher pH values by the reduction of O_2_ to OH^−^ and H^+^ to H_2_ [46]. In our experiment, pH increased with reducing distance to the periphyton surface or leaf surface (Figs 3, 5), while the estimated ORP by Nernst equation well matched the oppositely decreasing, and was also in accordance with the microprofiles in cyanobacterial granules [47]. Studies on the effect of submerged macrophytes on water ORP are relatively scarce. Preliminary studies have suggested that *P. crispus*, *Elodea canadensis*, and *Hydrilla verticillata* could decrease the ORP of bulk water [45], [48], [49]. To our knowledge, this is the first study that measured the ORP microprofiles of the stems and leaves of freshwater submerged macrophytes using microsensors, however, further studies are required to determine the dynamic mechanism of ORP in the microprofiles with a dense periphyton layer.

A natural phenomennon might influence the microprofiles in shallow freashwater lakes such as Taihu Lake (average depth, 1.9 m) is, the sedimentary resuspension due to stormy waves disturbance [50]. The deposition of suspended particulate solids on macrophyte surfaces can altered gas and nutrient exchanges by increasing the thickness of broad DBL, and reduced light transmission to the photosynthetically active leaf surfaces [51]. The consequent damages and decomposition were especially noted on mature and senescent leaves because a dense periphyton layer had already being formed gradually with aging in leaves [52], [53] which in the combination with deposited particles accelerated the decline of macrophytes.

The experiment was conducted under the similar conditions of *in situ* lake water, irradiance, and field temperature. Although it may be somewhat different from the field conditions, the present work revealed the micro-spatial variations in O_2_, pH, and ORP and their relationships with potential impact factors (periphyton, macrophyte growth, etc.). To date, microsensor is still an excellent tool for studying the microprofiles of submerged macrophytes with the high sensitivity and spatial resolution. In the future, the experiments should be conducted under the conditions of hydrodynamics, irradiation, and temperature similar to those in the field. Thus, the distributions of O_2_, pH, and ORP in the microprofiles may be close to the reality could be profiled to accurately reveal the microprofiles around submerged macrophytes.

## Conclusions

Microsensor profiling demonstrated that O_2_ concentration and pH increased whereas ORP decreased with reducing distance to the leaves of various ages and to the stem surface in *P. malaianus*. The fluctuation amplitudes of O_2_, pH, and ORP were the largest in the boundary layer of mature leaves and the lowest in senescent leaves. The periphyton increased the thickness of the broad DBL and fluctuation amplitudes of O_2_, pH, and ORP and negatively affected the growth of *P. malaianus*. Further studies are required to clarify the dynamics of O_2_, pH, and ORP in broad DBL aroung the *P. malaianus* in response to varied hydrodynamics, irradiance, temperature and nutrient conditions.
